# Ventilating the Nose

**Published:** 1898-10-22

**Authors:** 


### VENTILATING THE NOSE.

It is an old point, still it is worth hammering in, and Mr. Mayo Collier* has done well to insist on the importance of obtaining free ventilation of the nose in cases of aural disease. "Throat deafness" is, he says, well recognised, and is quite well understood, both within and outside the profession, to indicate a state of things which may be literally interpreted as Eustachian obstruction, the result of catarrh and swelling of the orifice of the Eustachian tube. But this is usually a sequela or extension of nasal catarrh, and consequently of nasal obstruction; indeed, simple obstruction of the orifice of one or both Eustachian tubes following nasal catarrh, and its accompanying nasal obstruction, is the starting-point or antecedent of a very large percentage of the commoner affections of the ear, which make themselves apparent to the patient as deafness, noises in the head, and "running" from the ear. In regard to prevention, treatment of the original nasal obstruction is all-important, but the same also holds good after aural disease is fully established. "Given chronic and well-marked affections of the middle ear set up and induced by an original Eustachian catarrh and obstruction, no permanent improvement can be hoped for or attained unless the *fons et origo mali*, the Eustachian obstruction, and its *causa causans*, the nasal obstruction, be removed." In treatment, then, as in prevention, the first step is to ventilate the nose. In many a case of common cold an astringent and antiseptic wash to the nose would restore nasal respiration, relieve Eustachian congestion and obstruction, and permit a free ingress of air to the tympanic cavity; while in chronic cases of deafness, with tinnitus and discharge due originally to Eustachian catarrh and obstruction, the same treatment applies, and is absolutely essential if any permanent improvement in such cases is to take place. The first step is to ventilate the nose. Whether this can be obtained by lotions, by the galvano cautery, or by other means is a detail, but only after ventilation of the nose is secured can the Politzer bag or the Eustachian catheter avail anything.

